# Trends in atmospheric concentrations of weed pollen in the context of recent climate warming in Poznań (Western Poland)

**DOI:** 10.1007/s00484-013-0781-5

**Published:** 2014-01-09

**Authors:** Paweł Bogawski, Łukasz Grewling, Małgorzata Nowak, Matt Smith, Bogdan Jackowiak

**Affiliations:** 1 Laboratory of Aeropalynology, Faculty of Biology, Adam Mickiewicz University, Umultowska 89, 61-614 Poznań, Poland; 2 Department of Climatology, Faculty of Geographical and Geological Sciences, Adam Mickiewicz University, Dzięgielowa 27, 61-680 Poznań, Poland; 3 Department of Dermatology, University of Medical Science, Przybyszewskiego 49, 60-355 Poznań, Poland; 4 Department of Oto-Rhino-Laryngology, Medical University of Vienna, Vienna, Austria

**Keywords:** Climate change, *Artemisia*, Poaceae, *Rumex*, Urticaceae, Weed plants, Phenology, Temperature

## Abstract

A significant increase in summer temperatures has been observed for the period 1996–2011 in Poznań, Poland. The phenological response of four weed taxa, widely represented by anemophilous species (*Artemisia* spp., *Rumex* spp. and Poaceae and Urticaceae species) to this recent climate warming has been analysed in Poznań by examining the variations in the course of airborne pollen seasons. Pollen data were collected by 7-day Hirst-type volumetric trap. Trends in pollen seasons were determined using Mann–Kendall test and Sen’s slope estimator, whereas the relationships between meteorological and aerobiological data were established by Spearman’s rank correlation coefficient. Significant trends in pollen data were detected. The duration of pollen seasons of all analysed taxa increased (from +2.0 days/year for Urticaceae to +3.8 days/year for *Rumex*), which can be attributed to a delay in pollen season end dates rather than earlier start dates. In addition, the intensity of *Artemisia* pollen seasons significantly decreased and correlates with mean July–September daily minimum temperatures (*r* = −0.644, *p* < 0.01). In contrast, no significant correlations were found between temperature and characteristics of *Rumex* pollen seasons. The results of this study show that observed shifts in weed pollen seasons in Poznań, i.e. longer duration and later end dates, might be caused by the recorded increase in summer temperature. This influence was the strongest in relation to *Artemisia*, which is the taxon that flowers latest in the year. The general lack of significant correlations between *Rumex* and Urticaceae pollen seasons and spring and/or summer temperature suggests that other factors, e.g. land use practices, could also be partially responsible for the observed shifts in pollen seasons.

## Introduction

Climate change is generally characterised by the increasing trends of average annual surface temperature, which in Europe exceeded 0.9 °C for the period 1901–2005 (Solomon et al. 2007). The average temperature for the European land area for the last decade (2002–2011) was 1.3 °C above the pre-industrial level, making it the warmest decade on record. Land temperatures, as well as the frequency and length of heat waves, is projected to increase over the twenty-first century (EEA 2012). It has been shown that the observed warming markedly affects the phenology of plants (Menzel et al. 2006; Gordo and Sanz 2009; Ziello et al. 2012a). Temperature increase is closely related to the longer growing season and advance of flowering time of plants (Walther et al. 2002). Some of these plants are of a great economic, ecological and health importance, and so it is essential to quantify any influence of climate variability on their phenophases (stage in a plant’s life cycle), such as budburst, fructification and flowering (Peñuelas and Filella 2001).

The latter can be determined both by in situ observations (Leon-Ruiz et al. 2011; Sparks et al. 2011; Lessard-Therrien et al. 2013) and by indirect methods, i.e. estimating the concentration of pollen grains in the atmosphere (Tormo et al. 2011; Jochner et al. 2012). Direct field observations supply precise information of phenological phases of particular species; however, they are often time consuming and based on a limited number of investigated specimens (Soudani et al. 2012). Conversely, pollen concentrations in the air reflect the flowering time of plants growing on a relatively large area around the pollen trap (Skjøth et al. 2010). Even though the aerobiological data series has some limitations, such as the possibility of being biased by long-distance transport of pollen (Skjøth et al. 2007; Tormo et al. 2011), it is still suitable to determining a generalised flowering phenophase in a region.

The main objective of this study was to examine the effect of recent temperature increases recorded in Poznań (Western Poland) between 1996 and 2011 on certain characteristics of the pollen seasons of weeds with the highest airborne pollen concentrations.

## Methods

### Study area and climate

**Fig. 1:**
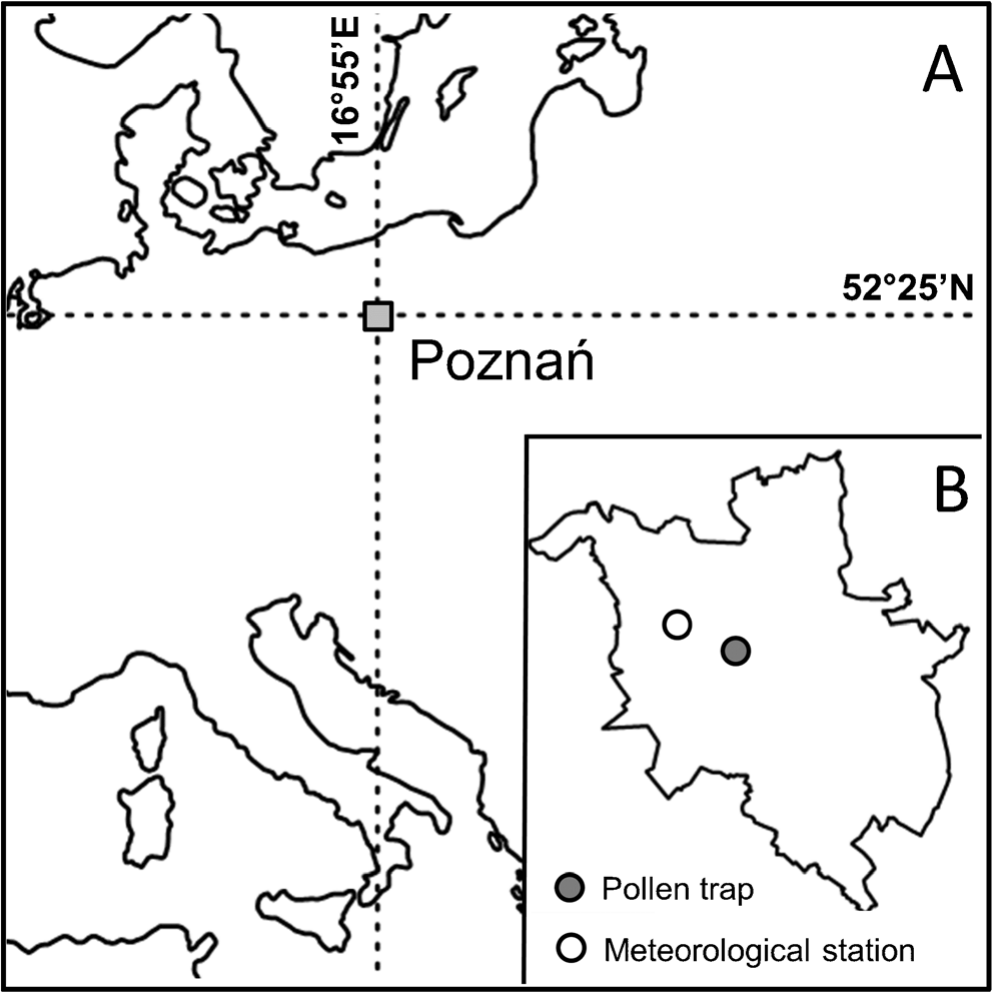
Location of Poznań in Europe (**a**) and pollen trap as well as meteorological station within Poznań administrative boundaries (**b**)

Poznań (50–150 a.s.l., 16°55′ E, 52°25′ N) is located in Western Poland (Wielkopolska province) with the population ~550,000 (CSO 2011). It has a temperate continental climate with cold winters and warm summers characterised by marked variability of weather phenomena. January is the coldest month of the year with the mean monthly temperature −1.6 °C, whereas July is the warmest one (18.1 °C). The mean annual precipitation is 517 mm (1951–2000 average) (Woś 2010). Daily mean, maximum and minimum temperatures were recorded at Ławica Airport (84 m a.s.l.), situated 4.25 km west of the pollen-monitoring site (Fig. 1), and supplied by the Institute of Meteorology and Water Management.

### Aerobiological data

Daily average pollen counts (1996–2011) were collected by 7-day volumetric spore trap of the Hirst design (Hirst 1952). The trap was located approximately 1 km from the city centre at the height of 33 m (Fig. 1). Two different pollen counting methods have been employed. From 1996 to 1999, the concentration of pollen grains in 1 m^3^ of air was calculated following the methods outlined by Stach (2000) where pollen grains were counted along 12 vertical transects. From 2000 to 2011, the four horizontal transects method recommended by Spanish Aerobiology Network was applied (Galán et al. 2007). The use of both vertical and horizontal transects has been shown to produce comparable results when similar percentages of the slide are examined (Comtois et al. 1999; Cariñanos et al. 2000). However, Kapyla and Penttinen (1981) stated that traverses along the length of the slide may give unreliable estimates because of the irregular transverse variation in the deposition of particles on the tape. The authors recommended that whole width of the tape should be studied because a considerable amount of particles were also found outside the 14 mm wide “effectively collecting area” below the orifice. Cotos-Yáñez et al. (2013) also reported that the distribution of pollen grains over vertical and horizontal transects is not uniform. However, it has also been shown that error is related to abundance of pollen grains on the slide, and the percentage of error diminishes as concentrations of pollen in the air increase (Tormo-Molina et al. 1996; Comtois et al. 1999). We examine weeds with the highest airborne pollen concentrations in Poznań, and so the use of different counting methods is not thought to influence the results of this study. The limits of pollen seasons were defined using the method proposed by García-Mozo et al. (2006) where the season start/end was determined as the day on which the count was higher than 1 pollen grain/m^3^, and that maintained itself through consecutive 5 days for start and proceeding 5 days for end of the pollen season. This method has already been used to calculate the limits of pollen seasons of weed species in Poznań (Stach et al. 2007; Alcázar et al. 2009). Due to trap failure during the 1998 pollen season, the dataset contained missing values, which were replaced by the mean daily average pollen count for that day (1996–2011 mean): 10 days, 22–31 July 1998.

Four weed taxa, i.e. *Artemisia* spp., *Rumex* spp. as well as Poaceae and Urticaceae species, were chosen for further analysis. From now on, *Artemisia* spp., *Rumex* spp. and Urticaceae and Poaceae species are simply referred to as *Artemisia*, *Rumex*, Urticaceae and Poaceae. The most widespread *Artemisia* species in the city are *A. vulgaris*, *A. absinthium* and *A. campestris* (Jackowiak 1993), which often invade areas of disturbed soils, roadsides and derelict wastelands (Maw et al. 1985; Barney and DiTommaso 2003). The most common species of Poaceae, *Rumex* and Urticaceae in Poznań and its surroundings were previously described by Stach et al. (2008) and Alcázar et al. (2009). From a phytosociological point of view, *Artemisia* spp., *Rumex* spp. and species from Urticaceae family are characteristic taxa within the class Artemisietea vulgaris (Matuszkiewicz 2006). The class comprises nitrophilous plants growing on ruderal areas. These habitats are common both inside and in the outskirts of Poznań (Adamczak 2008). The considerable area (26 %) covered by agricultural fields, wastelands and meadows in Poznań (MPU 2008) offers the habitats for the persistence of nitrophilous plant communities of the Artemisietea class.

In the study, we focused on examining the variation and trends of following characteristics of airborne pollen seasons:

1. Timing of pollen season, by investigating start, peak and end dates of the season; these data were converted to the day of the year from 1 January (DOY)
2. Intensity of pollen season by describing the seasonal pollen index (total amount of pollen during pollen season, SPI) and peak value (the highest daily pollen concentration)
3. Duration of pollen seasons.

### Statistical analysis

In aerobiological studies, the impact of meteorological factors, especially temperature, on pollen season parameters is often analysed (Emberlin et al. 2002; Grewling et al. 2012a; Ziello et al. 2012b). However, the temperature data usually originate from only one station, and it is possible to be biased by the changes in the measuring instruments, station relocation, urban growth (changes in immediate environment) or observation practices (Karl and Williams 1987; Peterson et al. 1998). To minimise these effects, the relative homogenisation approach is commonly applied (Vincent et al. 2002). It assumes that nearby located stations are exposed to almost the same climate character and their data could be used to build a reference series or to pairwise comparison with tested (candidate) series. As a result, it is possible to detect and remove inhomogeneities in temperature data (Venema et al. 2012).

The monthly mean daily average, maximum and minimum temperature series (from April to September) were tested in order to detect the possible data inhomogeneity. Meteorological data from surrounding stations, i.e. Leszno (70 km from Poznań), Piła (90 km) and Gorzów Wielkopolski (130 km) were used as a reference series. The temperature data were significantly correlated (*r* = 0.80–0.94, *p* < 0.01, data not shown) with the data from Poznań and with each other. Therefore, these series were used to perform the tests of data homogeneity:

1. Standard normal homogeneity test (SNHT) for shift to detect a sudden change in mean value of meteorological parameters (Alexandersson 1986)
2. SNHT for trends to detect a graduate, artificial change (Alexandersson and Moberg 1997)
3. Bivariate test (Potter 1981).

For the trend analysis, only homogenous series of monthly temperatures were taken into account. The evaluation of the statistical significance and the slope of the existing trend the non-parametric Mann–Kendall test and Sen’s method have been used (Mann 1945; Kendall 1975; Salmi et al. 2002). The relationships between pollen season parameters and air temperature have been examined by Spearman’s rank correlation coefficient. Only monthly temperature data that could potentially affect pollen season characteristics have been taken into account; for instance, meteorological data recorded before and during the pollen season were correlated with its duration and intensity, whereas pollen season start, peak and end dates were linked with temperature data recorded only before these aerobiological parameters. The analyses have been executed in the XLSTAT software (Addinsoft™, SARL), and in the MAKESENS application (Version 1.0 Freeware, Copyright Finnish Meteorological Institute). Creating reference series and the relative homogenisation testing of data have been conducted in ProClimDB and Anclim software packages (Stepanek 2008a, b).

## Results

### Meteorological data

**Fig. 2:**
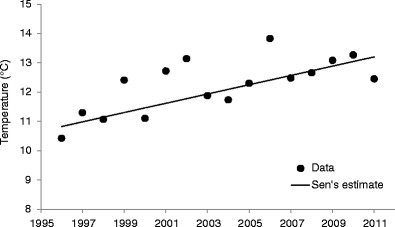
Trend in mean minimum July–September temperature in Poznań (1996–2011). Mann–Kendall trend test (test *Z* column), Sen’s slope estimation (*Q* column), and the limits of confidence interval (95 %: *Q*_min95_ and *Q*_max95_) are presented in Table 2

Generally, for the period 1996–2011, the monthly temperature data series were homogeneous, although, in June, both SNHT and bivariate tests showed that there was an inhomogeneity in the mean daily maximum and minimum temperatures (Table 1). These two temperature series were rejected from further analyses. Mann–Kendall test and Sen’s method revealed existence of six statistically significant temperature trends (Table 2; Fig. 2). All detected series were characterised by a significant temperature increase and the most distinct one was observed during the July–September period (+0.16 °C/year, *p* < 0.01).

**Table 1:** Results of homogeneity testing

Month	Homogeneity test								
	SNHT shift			SNHT trend			Bivariate test		
	*T*_min_	*T*_max_	*T*_mean_	*T*_min_	*T*_max_	*T*_mean_	*T*_min_	*T*_max_	*T*_mean_
April	5.07	2.67	1.66	1.56	2.33	1.34	6.11	2.66	2.49
May	2.32	3.00	4.33	1.77	0.60	3.16	3.34	3.97	5.59
June	7.00*	9.22*	3.69	5.56	8.37*	3.59	7.42*	9.85*	6.28
July	4.56	5.96	5.40	4.32	6.05	4.60	6.26	6.18	6.41
August	1.66	2.32	6.14	1.34	1.98	5.01	2.88	2.75	6.65
September	3.57	2.50	5.06	4.31	2.80	4.70	5.77	5.90	4.88

Average minimum, maximum and mean monthly temperature series tested with three kinds of relative homogeneity test

*p < 0.05

**Table 2:** Significant temperature trends in Poznań (1996–2011) calculated from homogeneous series: Mann–Kendall trend test (test *Z* column), Sen’s slope estimation (*Q* column) and the limits of confidence interval (95 %: *Q*_min95_ and *Q*_max 95)_

Temperature series	Test *Z*	*Q*	*Q*_min95_	*Q*_max95_
*T*_min_ July (°C)	2.026*	0.18	0.004	0.372
*T*_min_ July–September (°C)	2.746**	0.16	0.063	0.209
*T*_min_ July–August (°C)	2.476*	0.15	0.056	0.246
*T*_mean_ June–July (°C)	2.566*	0.18	0.030	0.297
*T*_mean_ April–July (°C)	2.026*	0.10	0.004	0.204
*T*_mean_ April–September (°C)	2.116*	0.12	0.015	0.198

*p < 0.05; **p < 0.01

### Aerobiological data

#### Timing of pollen seasons

Amongst investigated taxa, the Poaceae pollen season started the earliest (mean = 131 DOY, 11th of May) and became significantly earlier during the studied period (−1.03 day/year, *p* < 0.05) (Tables 3 and 4). Urticaceae pollen seasons usually started in the second fortnight of May, whereas the onset of *Artemisia* pollen season occurred markedly later (middle of July) compared to other taxa. Similarly to Poaceae, the *Artemisia* pollen seasons in Poznań started significantly earlier (−0.75 day/year, *p* < 0.01) and were strongly correlated with mean July daily minimum temperatures (*r* = −0.805, *p* < 0.01) (Table 5).

**Table 3:** Descriptive statistics of pollen season parameters in Poznań, 1996–2011

Taxon	Descriptive statistics	Characteristics of pollen season					
		Start date (DOY)	Peak date (DOY)	End date (DOY)	Duration (days)	Peak value (P/m^3^)	SPI (pollen)
Poaceae	Max	149	194	273	148	524	7,487
	Min	116	149	230	100	108	2,272
	Mean	131	177	256	124	279	4,100
	SD	8.9	11.9	13.4	17.1	116.2	1,368.3
	CV (%)	6.8	6.7	5.2	13.7	41.6	33.4
*Rumex* spp.	Max	150	200	273	145	153	2,496
	Min	119	131	206	83	22	282
	Mean	134	169	249	115	51	1,147
	SD	7.8	18.9	19.0	20.8	31.6	462.6
	CV (%)	5.8	11.2	7.6	18.0	62.1	40.3
Urticaceae	Max	160	227	274	149	1080	22,414
	Min	120	176	240	87	162	662
	Mean	147	203	261	114	534	10,197
	SD	11.6	13.4	11.3	16.9	312.2	5,728.9
	CV (%)	7.9	6.6	4.3	14.8	58.5	56.2
*Artemisia* spp.	Max	208	228	271	81	439	3,429
	Min	189	209	241	36	35	369
	Mean	198	219	253	56	137	1,421
	SD	5.0	4.8	11.7	15.1	104.1	809.0
	CV (%)	2.5	2.2	4.5	27.0	75.9	56.9

**Table 4:** Mann–Kendall trend test (test *Z* column) and Sen’s slope estimate (*Q* column) of pollen season parameters of weed taxa

Pollen season parameter	Poaceae		*Rumex* spp.		Urticaceae		*Artemisia* spp.	
	Test *Z*	*Q*	Test *Z*	*Q*	Test *Z*	*Q*	Test *Z*	*Q*
Start date (DOY)	−2.12*	−1.03	−0.59	−0.31	−1.04	−0.39	−2.86**	−0.75
End date (DOY)	2.7**	1.86	2.66**	2.81	2.60**	1.41	2.26*	1.27
Peak date (DOY)	−0.27	−0.22	1.40	1.56	−1.22	−0.79	0.46	0.10
Duration (days)	3.52**	3.00	2.75**	3.76	2.12*	2.00	2.75**	2.49
Peak value (P/m^3^)	−0.81	−6.56	−1.67	−1.50	0.41	7.96	−1.76	−7.32
SPI (pollen)	−0.41	−58.5	−1.22	−11.75	1.49	517.17	−2.12*	−81.33

*p < 0.05; **p < 0.01

**Table 5:** Spearman correlation coefficients between the pollen season parameters and homogenous monthly temperature series recorded in Poznań (1996–2011)

Taxon	Pollen season parameter	*T*_mean_ April–July	*T*_mean_ April–September	*T*_mean_ June–July	*T*_min_ July	*T*_min_ July–August	*T*_min_ July–September
Poaceae	Start date	–	–	–	–	–	–
	Peak date	−0.625*	–	ns	ns	–	–
	End date	ns	ns	ns	0.515*	0.627*	0.670**
	Duration	ns	ns	0.536*	0.649**	0.780**	0.830**
	Peak value	−0.631*	ns	ns	ns	ns	ns
	SPI	−0.632*	−0.553*	ns	ns	ns	ns
*Rumex* spp.	Start date	–	–	–	–	–	–
	Peak date	ns	–	ns	ns	–	–
	End date	ns	ns	ns	ns	ns	ns
	Duration	ns	ns	ns	ns	ns	ns
	Peak value	ns	ns	ns	ns	ns	ns
	SPI	ns	ns	ns	ns	ns	ns
Urticaceae	Start date	–	–	–	–	–	–
	Peak date	−0.512*	–	ns	ns	ns	–
	End date	ns	ns	ns	ns	ns	ns
	Duration	ns	ns	ns	ns	ns	ns
	Peak value	ns	ns	ns	ns	ns	ns
	SPI	ns	ns	ns	ns	ns	ns
*Artemisia* spp.	Start date	−0.562*	–	−0.728**	−0.805**	–	–
	Peak date	ns	–	ns	ns	ns	–
	End date	ns	ns	ns	0.552*	0.565*	0.626*
	Duration	0.532*	0.691**	0.686**	0.732**	0.670**	0.750**
	Peak value	ns	ns	−0.629*	−0.547*	ns	−0.635**
	SPI	ns	ns	−0.571*	−0.521*	−0.615*	−0.644**

*ns* not significant

*p < 0.05, **p < 0.01

The days with maximum daily pollen level were usually recorded a few weeks after pollen season start dates (from 3 to 8 weeks for *Artemisia* and Urticaceae, respectively) (Table 3). The peak dates of Poaceae and Urticaceae pollen seasons were significantly (*p* < 0.05) correlated with mean April–July daily average temperatures (*r* = −0.625 and *r* = −0.512, respectively), but no statistically significant shifts in this pollen season parameter were noticed (Tables 4 and 5).

The mean values of pollen season end dates were very similar within the investigated taxa, and occurred in the first fortnight of September (Table 3). Interestingly, significant trends (*p* < 0.05) in pollen season end dates were found for all examined taxa (ranging from +1.27 to +2.81 days/year for *Artemisia* and *Rumex*, respectively) (Table 4). End dates of *Artemisia* and Poaceae pollen seasons were significantly correlated with mean July–September daily minimum temperatures (*r* = 0.670, *p* < 0.01 and *r* = 0.626, *p* < 0.05). In contrast, no statistically significant correlations were found between selected meteorological parameters and pollen season end dates of *Rumex* and Urticaceae (Table 5).

#### Intensity of pollen seasons

The highest sum of daily average pollen concentration recorded in the season was for Urticaceae (mean = 10197 pollen), whereas the lowest was for *Rumex* (mean = 1147 pollen) (Table 3). The pollen seasons of examined taxa in Poznań generally have a tendency towards decreasing intensity, but the only significant trend was for *Artemisia* pollen seasons (−81.3 pollen/year, *p* < 0.05) (Table 4). The exception was Urticaceae, where a slight increase in SPI was recorded (not significant). The SPI and peak value of *Artemisia* were strongly affected by mean July–September daily average temperatures (*r* = −0.644, *p* < 0.01 and *r* = −0.635, *p* < 0.01, respectively). The grass pollen seasons intensity showed significant relationships with mean April–July daily average temperatures (*r* = −0.632, *p* < 0.05). The same meteorological conditions also influenced the peak daily values of Poaceae (*r* = −0.631, *p* < 0.05). No significant correlations were found between *Rumex* and Urticaceae pollen season intensity and selected weather parameters (Table 5).

#### Duration of pollen seasons

Compared to *Artemisia* (mean pollen season duration = 56 days), the pollen seasons of Poaceae, *Rumex* and Urticaceae were relatively long (>100 days) (Table 3). Significant (*p* < 0.01) lengthening of pollen seasons were seen for all examined taxa and ranged from +2.0 (Urticaceae) to +3.8 (*Rumex*) days/year (Table 4). The results of correlation analysis revealed significant (*p* < 0.01) positive relationships between mean July–September daily minimum temperatures and duration of *Artemisia* and Poaceae pollen seasons (*r* = 0.750 and *r* = 0.830, respectively). Similarly to pollen season intensity, no significant correlations were found between *Rumex* and Urticaceae pollen season duration and selected meteorological parameters (Table 5).

## Discussion

In order to determine whether data variability is only caused by climate change, it is necessary to analyse homogenised time series. On average, one inhomogeneity is detected per 15–20 years in climatological data series (Venema et al. 2012). After homogeneity testing of mean monthly minimum, maximum and average temperature data recorded in Poznań, six trends in spring and summer temperatures have been identified as an effect of climate change. A number of significant trends in the characteristics of pollen seasons of anemophilous weed plants were simultaneously found in Poznań.

### Timing of pollen seasons

The strongest shifts were related to *Artemisia* and Poaceae pollen seasons that started earlier and ended significantly later than they did in the mid-1990s. The observed advance of *Artemisia* pollination period (−0.75 day/year, *p* < 0.01) continues the tendency described in Poznań for the years 1995–2004 (Stach et al. 2007). It is worth noticing that *Artemisia* pollen season start dates significantly correlated with mean daily minimum temperatures in July (*r* = −0.805, *p* < 0.01), which significantly increased in Poznań over the same period (+0.18 °C/year, *p* < 0.05). This observation agrees with previous studies (von Wahl and Puls 1989; Wolf et al. 1998) showing that high minimum temperatures accelerate the rate of development of *Artemisia* inflorescences and the release of its pollen. On the other hand, regional-scale studies showed that *Artemisia* pollen seasons were notable delayed in areas where the mean June–July daily minimum temperatures exceeded 13 °C (Grewling et al. 2012b).

A previous study conducted in Poznań (Stach et al. 2008) showed that the onset of Poaceae pollen seasons was negatively affected (*p* < 0.01) by temperature recorded before pollen season start dates, particularly mean March–May daily average temperatures. However, due to the lack of a significant trend in temperature data during early spring, we did not investigate the relationship between temperature and Poaceae pollen season start dates. The onset of *Rumex* and Urticaceae pollen seasons also advanced in Poznań, but the observed trends were not statistically significant. In general, earlier start dates of pollen seasons of weed plants was recorded across Europe during the last decades (Frenguelli 2002; Van Vliet et al. 2002; Clot 2003; García-Mozo et al. 2010a; Makra et al. 2011), and most of this changes might be related to the recent temperature increase. In some regions, e.g. in Southern Europe, these tendencies are not so distinct and in a few cases the delay of pollen seasons was noticed (Damialis et al. 2007; Recio et al. 2009; Recio et al. 2010; Tormo-Molina et al. 2010) and could be linked to the influence of water availability on plant growth and development in these regions (García-Mozo et al. 2009, García-Mozo et al. 2010b; Recio et al. 2010). It should therefore be noted that Wielkopolska is one of the driest regions in Poland (Woś 2010) and further shortage of rainfall, as projected for Central Europe (EEA 2012), might possibly affect the course of pollen seasons. However, no significant decreases in rainfall were recorded during the studied period, and as a result, this parameter was not considered to be responsible for the temporal changes in weed pollen seasons in Poznań.

### Intensity of pollen seasons

The *Artemisia* pollen index significantly decreased in Poznań. A similar trend was noticed in Szczecin, located around 200 km North-West from Poznań (Puc and Wolski 2013) as well as in many sites in Europe (Ziello et al. 2012b). Our study revealed that *Artemisia* pollen season intensity was significantly negatively correlated with minimum temperatures during the summer months. These results generally agree with studies performed by Munuera Giner et al. (1999) who concluded that, due to the high summer temperatures, plants may allocate their resources into vegetative organs rather than into generative ones. In addition, increasing temperatures stimulate evaporation and so limits the amount of water available for plants (Zhao and Running 2010). A reduction in reproductive organs and the subsequent decrease of pollen production is one of the ways for limiting water losses (Tardieu 2013). Low-magnitude *Artemisia* pollen seasons have already been linked with high temperatures and scarce rainfall in previous weeks (Munuera Giner et al. 1999; Stach et al. 2007).

In contrast, the seasonal sum of pollen of other taxa did not change significantly in Poznań. The intensity of Poaceae and *Rumex* pollen seasons slightly decreased, whereas Urticaceae showed a tendency towards more intense seasons, but the trend was not significant. Interestingly, during the previous study conducted in Poznań (Alcázar et al. 2009), a clear trend towards declining annual pollen counts of *Rumex* and Urticaceae was recorded over 1995–2005 study period. This contradiction is understandable by examining the SPI during the period 2007–2011. This is because four of the last five pollen seasons, with a SPI > 14,000 pollen, rank among the most intense Urticaceae pollen seasons in the aerobiological record in Poznań (data not shown). It is unlikely that such strong increases in SPI are linked to favorable weather conditions, as only weak and not significant relationships were found between SPI of Urticaceae and temperature. It is therefore suggested that these variations in pollen season intensity could be modified by factors other than climate, e.g. by land-use practices.

For instance, Spieksma et al. (2000) proved that cutting *Artemisia* nearby the pollen trap can markedly reduce the pollen concentration on the street level. The importance of land use changes and changes in management practices has also been linked to decreasing Poaceae pollen counts, such as urbanisation reducing the amount of meadows and green areas and more frequent mowing (Stach et al. 2008; Alcázar et al. 2009; Puc and Wolski 2013). In addition, Ziello et al. (2012b) reported that the significant decreases for Amaranthaceae and *Artemisia* pollen season intensity could be possibly explained by intensification of weed control and less agricultural land set aside in the context of increasing bioenergy demand. Unfortunately, it is difficult to evaluate the influence of land-use practices in modifying the long-term changes in the intensity of pollen seasons without detailed analysis of land cover data as in the study of grass pollen counts in North London conducted by Emberlin et al. (1993). This, as well as the effect of other factors potentially responsible for the fluctuation in annual pollen sums, such as temporal variation in the North Atlantic Oscillation (Smith et al. 2009) and increasing level of CO_2_ (Ziska and Caulfield 2000; Ziska and Beggs 2012), was beyond the scope of this study.

### Duration on pollen seasons

According to the Report of World Health Organization (Huynen et al. 2003), climate warming is likely to extend the duration of pollination periods of grasses and weeds. Our study concurs with this projection as significantly longer pollen seasons were seen for all examined taxa that positively correlated with increasing summer temperatures (significant for *Artemisia* and Poaceae). The observed lengthening of pollen seasons varied from +2.0 days/year for Urticaceae to +3.8 days/year for *Rumex*. Longer pollination periods of weed plants in Poznań can be attributed to the delayed pollen season end dates rather than advanced start dates. Ziska et al. (2011) showed that the increase of ragweed (*Ambrosia* spp.) pollen seasons in North America was primarily associated with a delay in first frost of the autumn season and lengthening of the frost free period. Similarly, rising temperatures observed in Poznań during late summer (August–September) may increase the period for plant development and pollen release. Pollen seasons of weed plants generally last longer and end later in Southern Europe compared to Central Europe, which can be attributed to more favourable weather conditions and the presence of a larger number of species (Spieksma et al. 1993; Alcázar et al. 2009). For instance, in the countries south of the Carpathian Mountains, i.e. in Hungary, Serbia and Bulgaria, late-flowering *Artemisia* species are responsible for the second peak of pollen concentrations that usually lasts till the end of September (Grewling et al. 2012b). Currently, the “autumn” peak of *Artemisia* pollen is rarely recorded in Poland. However, climate warming may affect migration and distribution of species (Hyvönen et al. 2011; Chuine et al. 2012) and, as a result, the composition of plant communities (Walther et al. 2002; Munson et al. 2012). These spatial changes in the long term may influence temporal variations of pollen seasons and therefore should be considered in future studies.

## Conclusions

Pollen seasons of anemophilous weed taxa in Poznań changed over the 1996–2011 period. Pollination periods now start earlier for *Artemisia* and Poaceae, end later for *Artemisia*, Poaceae, *Rumex* and Urticaceae as well as last longer for the four taxa studied. The intensity of *Artemisia* pollen seasons were also found to be significantly decreasing. Of all these changes, only those of Poaceae and *Artemisia* were significantly correlated with recorded increases in summer temperatures, suggesting that climate warming is the main factor responsible for the observed shifts. The general lack of significant correlations between *Rumex* and Urticaceae pollen seasons and spring or summer temperature suggests that other factors, e.g. land use practices, could be also partially responsible for observed shifts in pollen seasons.
